# Risk Factors for Neonatal Mortality at the Yaounde Gynaeco-Obstetric and Pediatric Hospital, Cameroon

**Published:** 2014-06-29

**Authors:** Andreas Chiabi, Vanessa Takou, Evelyn Mah, Seraphin Nguefack, Hypolyte Siyou, Virginie Takou, Pierre-Fernand Tchokoteu, Elie Mbonda

**Affiliations:** Yaounde Gynaeco-Obstetric and Pediatric Hospital, Faculty of Medicine and Biomedical Sciences, University of Yaounde I, Cameroon

**Keywords:** Neonatal Mortality, Hospital, Risk Factors, Cameroon

## Abstract

***Objective:*** Neonatal mortality is a major health problem in sub-Saharan Africa and the risk factors are not well established. The objective of this study was to determine the risk factors for neonatal mortality at the Yaounde Gynaeco-Obstetric and Pediatric Hospital.

***Methods:*** We conducted a retrospective and analytic case-control study from the medical records of newborns admitted at the neonatal unit of this hospital between 1^st^ March 2003 and 31^st ^December 2012. 850 subjects were enrolled; that is 425 cases and 425 controls.

***Findings*** ***:*** The intra-hospital neonatal mortality rate was 9.83%. The main causes of neonatal mortality were in descending order: neonatal sepsis (60.2%), complications from prematurity (42.6%), birth asphyxia (37.4%), and congenital malformations (11.8%).The most prominent risk factors for neonatal mortality after multivariate analysis with logistic regression were: prolonged membrane rupture (OR: 3.8719, 95% CI: 2.3619-6.3471; *P=*0.0000), low birth weight (OR: 1.6240, 95% CI: 1.0108-2.6091; *P*=0.0450), Apgar score less than 7 at the 5th minute (OR: 6.8979, 95% CI: 4.0709-11.6883; *P*=0.0000), and congenital malformations (OR: 4.3307, 95% CI: 1.6120-11.6347; *P*=0.0037). Delivery by cesarean section (OR: 0.2644, 95% CI: 0.1478-0.4732;* P*=0.0000) and being born in this hospital (OR: 0.4409;95% CI: 0.2566-0.7576;* P*=0.0030) were protective.

***Conclusion:*** Neonatal mortality was influenced by both maternal and neonatal factors. This could be reduced through sensitization of pregnant women on the need of good quality antenatal visits, and capacitating the health personnel on the adequate management of high risk neonates.

## Introduction

The World Health Organization (WHO) estimates that more than 3 million neonates worldwide die within the first months of life, with a similar number of stillbirths. During the first month of life, more than a quarter of these deaths occur during the first 24 hours of life, and 75% within the first week^[^^1^^]^. In 2010, the global neonatal mortality rate (NMR) was 23‰, and the highest rates of 33‰ and 35‰ were observed in South Asia and sub-Saharan Africa respectively^[^^2^^]^. However, the global NMR worldwide over the years has been: 28‰ in 2006^[^^3^^]^, 30‰ in 2007^[^^4^^]^, 24‰ in 2009^[^^5^^]^ and 23‰ in 2010^[^^2^^]^. In Cameroon in 2011, the NMR was 31‰ and approximately one out of eight children died before reaching the age of five^[^^6^^]^.

 In Benin the NMR was 55.6‰ in 1991^[^^35^^]^. In Nigeria, the NMR ranged between 25.6/1000 live births and 72.4/1000 live births in out-born neonates, and 336.5/1000 live births for out-borns^[^^36^^]^. An early neonatal mortality rate of 68/1000 live births and 16/1000 live births in Kenya ^[^^41^^]^ and Ghana respectively.

 Mortality in hospitalized neonates also vary from country to country: 13.1%, 28.3%, 26.5%, 34.2% in Burkina Faso, Togo, Ivory Coast, Guinea Conakry respectively ^[^^37^^,^^38^^,^^39^^,^^40^^]^.

 The objective of this study was to determine the socio-demographic, maternal, obstetric and neonatal risk factors associated with neonatal mortality in this setting, which will serve as a basis in proposing interventions to curb it.

## Subjects and Methods

It was a retrospective and analytic case-control study with data collected from medical records of newborns admitted in the neonatal unit (NNU) of the Yaounde Gynaeco-Obstetric and Pediatric Hospital (YGOPH) between 1^st^ March 2003 and 31^st^ December 2012 (9 years 10 months). 

 Enrolled in this study were all newborns admitted in the NNU of this hospital, and who died during hospitalization within 28 days of life. Newborns admitted in this unit and discharged alive after management were the controls. The cases and controls were matched for gestational age±2 weeks of amenorrhea. Excluded from the study were neonates admitted during the study period but who died or were discharged alive after 28 days of life, and also files with insufficient data.

 Data extracted from these records were put on questionnaires. The variables collected were: 

- For the mother: age, place of residence, marital status, level of education, occupation, gender, past history of prematurity, abortions and/or stillbirths, neonatal deaths, follow-up of pregnancy, pathologies during pregnancy, perinatal fever, time of rupture of membranes with respect to delivery, nature of the amniotic fluid, place of birth, gestational age and mode of delivery.

- For the newborn: the sibling rank, age at admission, the number of fetuses, sex, Apgar score at the 5th minute, birth weight, and pathologies presented during admission. 

 Data were entered and analyzed in the Epi-Info Version 3.5.3software, and the Fisher's exact test was used to compare proportions. The statistical significance level was *P* value <0.05. The Odds ratio (OR) with its confidence interval (CI) at 95% were used to assess the risk of an association. The factors significantly associated with neonatal mortality in the bivariate analysis further underwent multivariate analysis with logistic regression.

 Ethical clearance was obtained from the Ethics Committee of the hospital, and the data were kept confidential.

## Findings

*Study population: *During the study period, 7824 neonates were admitted to the NNU of the YGOPH. From this number, 769 died and 7,055 were discharged alive, giving an intra-hospital neonatal mortality of 9.83%. For the study, we selected 425 cases and 425 controls, who met up with our inclusion criteria giving a case-control ratio of 1:1. 

 Of the 425 neonates who died, 233 (54.8%) were males and 192 (45.2%) females, giving a sex ratio of 1.21, with no statistically significant difference (*P=*0.2). Most neonates who died, 397 (93.4%) were admitted during the early neonatal period (≤7 days), against 28 (6.6%) in the late neonatal period (>7 days) (*P=*0.08). Most deaths occurred in the early neonatal period, 307 (72.2%) against 118 (27.8%) in the late neonatal period. Amongst the 425 neonates who died, 77 (18.1%) died during the first 24 hours of life. The mean duration of hospitalization of the neonates who died was about 5 days (range 1- 27 days). 

 Half of these newborns, 221 (52%) had a low birth weight (<2500g) and 15 (3.5%) were macrosomic (> 4000 g). The mean gestational age of the newborns who died was 35 weeks of amenorrhea, with extremes of 22 and 44 weeks. Nearly half of the newborns, 205 (48.2%) who died were born at term (37 to 42 weeks of amenorrhea); 201 (47.3%) neonates came from the YGOPH, while 224 (52.7%) came from elsewhere (20% from other referral hospitals, 16.9% from other health facilities, and 15.8% from home) (Table 1). 

**Table 1:** Characteristics of the dead neonates

		**n**	** (%)**
**Sex**	**Males**	233	54.8
	**Females**	192	45.2
**Period of admission (days)**	**≤ 7**	397	93.4
	**> >7**	28	6.6
**Birth weight (g)**	**< 2500**	221	52
	**2500-4000**	189	44.5
	**≥4000 **	15	3.5
**Gestational age (weeks)**	**< 37**	200	47.1
	**37-42 **	205	48.2
	** ≥42**	20	4.7
**Origin of the neonates**	**YGOPH**	201	47.3
	**Others** *	224	52.7

* other hospitals: 20%; peripheral health centers: 16.9%; home: 15.8%

*Socio-demographic characteristics of the mothers:* Celibacy was a risk factor for neonatal death (OR: 1.65; 95%CI: 1.19-2.27; *P*=0.001) and being married was a protective factor (OR: 0.62; 95% CI: 0.45-0.86; *P*=0.002). Primary or secondary level of education was a risk factor (OR: 1.43; 95%CI: 1.01-2.02; *P*=0.03) (Table 2). 

*Obstetrical characteristics:* Among the analyzed obstetrical factors, primiparity was the only risk factor (OR: 1.51; 95%CI: 1.14-2.01;* P*=0.002) (Table 3). 

*Maternal illnesses:* Amongst the 127 (30.8%) mothers of dead neonates who had had at least one acute or chronic illness and/or pathology during pregnancy, the most common pathology encountered was malaria in 59 (46.5%) of the mothers. None of the maternal pathologies or disorders had a significant impact on neonatal mortality.

*Maternal factors associated with labor and delivery:*

Perinatal fever (OR: 2.09; 95%CI: 1.28-3.43; *P*=0.002), prolonged rupture of membranes ( 12 hours before delivery) (OR: 2.70; 95% CI: 1.99-3.64;* P<*0.001), meconium stained amniotic fluid (OR: 2.33; 95% CI: 1.59-3.43; *P<*0.001), delivery out of a hospital setting (OR: 2.17; 95%CI: 1.01-4.66; *P*=0.03) were significant risk factors associated with neonatal mortality; whereas cesarean section (OR: 0.31, 95% CI: 0.22-0.42; *P*< 0.001) was a protective factor (Table 4).

*Characteristics associated with neonates: *Provenance of the neonates from the YGOPH was a protective factor against neonatal mortality (OR: 0.35; 95%CI: 0.26-0.47:* P*<0.001). First borns (OR: 1.48, 95%CI: 1.12-1.96: *P*=0.003), Apgar score less than 7 at the 5th minute (OR: 3.66; 95%CI: 2.68-4.98;* P*<0.001), and low birth weight (<2500g) (OR: 1.43, 95%CI: 1.09-1.88; *P*=0.005) were risk factors (Table 5). 

**Table 2:** Distribution of the neonates according to maternal socio-demographic factors

**Parameter**		**Cases**	**Control**	**OR**	**CI 95%**	***P. *** **value**
		**n (%)**	**n (%)**			
**Maternal age range ** **(ye) ** **n** a **=796 (90+706)**	**< 20**	48 (53.3)	42 (46.7)	1.11	0.73 - 1.77	0.34
	**≥ 20**	353 (50)	353 (50)			
**Place of residence ** **n** a **= 612 (93+519)**	**Rural**	51 (54.8)	42 (45.2)	1.27	0.81 - 1.97	0.29
	**Urban**	254 (48.9)	265 (51.1)			
**Marital status ** **n** a **= 609 (296+313) and ** **(311+298)**	**Single **	170 (57.4)	126 (42.6)	1.65	1.19 - 2.27	0.001
	**Others** b	141 (45.0)	172 (55.0)			
	**Married**	141 (45.3)	170 (54.7)	0.62	0.45 - 0.86	0.002
	**Others** c	170 (57.0)	128 (43.0)			
**Educational level ** **n** a **= 557 (352+205)**	**Primary/Secondary**	198 (56.3)	154 (43.7)	1.43	1.013 -2.02	0.026
	**University**	97 (47.3)	108 (52.7)			
**Occupation ** **n** a ** = 660 (92+568)**	**Salaried **	42 (45.7)	50 (54.3)	0.78	0.50 - 1.21	0.2
	**Others** d	295 (51.9)	273 (48.1)			

a number of informative files,

b not single (married, widow, divorced),

c not married (single, widow, divorced),

d (private sector, students, housewives, farmers, traders, unemployed); OR: Odds Ratio; CI: Confidence interval

**Table 3:** Distribution of neonates according to maternal obstetrical factors

**Parameter**		**Cases**	**Control**	**OR**	**CI 95%**	***P. *** **value**
		**n (%)**	**n (%)**			
**Parity ** **n** a **=803 (346+457)**	**Primiparous**	194 (56.1)	152 (43.9)	1.51	1.14 - 2.01	0.002
	**Others**	209 (45.7)	248 (54.3)			
**PH of prematurity ** **n** a **= 802 (23+779)**	**Yes **	10 (43.5)	13 (56.5)	0.76	0.33 - 1.75	0.3
	**No**	392 (50.3)	387 (49.7)			
**PH of still birth or miscarriage ** **n** a **=802 (271+531)**	**Yes **	124 (45.8)	147 (54.2)	0.77	0.57 - 1.03	0.04
	**No**	278 (52.4)	253 (47.6)			
**PH of neonatal death ** **n** a **= 802 (48+754)**	**Yes**	24 (50.0)	24 (50.0)	0.99	0.55 - 1.78	0.5
	**No**	378 (50.1)	376 (49.9)			
**No of antenatal consultations ** **n** a **=411 (141+270)**	**< 4**	81 (57.4)	60 (42.6)	1.31	0.87 - 1.97	0.1
	**≥ 4**	137 (50.7)	133 (49.3)			
**Pathologies during pregnancy ** **n** a **=830 (260+570)**	**Yes **	127 (48.8)	133 (51.2)	0.95	0.71 - 1.28	0.4
	**No**	285 (50.0)	285 (50.0)			

a number of informative files; PH: Past history; OR: Odds Ratio; CI: Confidence interval

*Neonatal pathologies on admission: *Neonatal sepsis (OR: 1.35; 95%CI: 1.02-1.77; *P*=0.02), neonatal asphyxia (OR: 3.25, 95%CI: 2.34-4.51; *P*<0.001), and congenital malformations (OR: 4.59; 95%CI: 2.41-8.75; *P*<0.001) were statistically associated with neonatal mortality (Table 6).

*Causes of neonatal deaths: *The main causes of neonatal deaths were: neonatal sepsis 256 (60.2%), complications from prematurity181 (42.6%), neonatal asphyxia 159(37.4%), and congenital malformations 50 (11.8%). The most common malformations found in 50 neonates were polymalformative syndromes (18%), congenital heart disease (12%), esophageal atresia (10%), laparoschisis (10%), and meningo-encephalocele (10%).

 On multivariate analysis with logistic regression of the significant risk factors above, prolonged membrane rupture (OR: 3.87, 95%CI: 2.36-6.35; *P*<0.001), low birth weight (OR: 1.62, 95%CI: 1.01-2.61); *P*=0.04), Apgar score less than 7 at the 5th minute (OR: 6.90, 95%CI: 4.07-11.69; *P*<0.001), and congenital malformations (OR: 4.33, 95%CI: 1.61-11.63; *P*=0.004), persisted as the risk factors for neonatal mortality. Cesarean section (OR: 0.26, 95%CI: 0.15-0.47;* P*<0.001) and being born at the YGOPH (OR: 0.44; 95% CI: 0.26-0.76;* P*=0.003) remained protective factors.

## Discussion

The study matched 425 neonates who died in the NNU of the YGOPH, with 425 controls discharged alive from same unit after management. After multivariate analysis, prolonged rupture of the membranes, low birth weight, congenital malformations and a low Apgar score represented risk factors for neonatal deaths.

**Table 4:** Distribution of factors associated with labor and delivery

**Parameter **			**Cases**		**Controls**	**OR**	**CI 95%**	***P. *** **value**
			**n (%)**		**n (%)**			
**Perinatal fever ** **n** a **= 850 (77+773)**		**Yes **	51 (66.2)		26 (33.8)	2.09	1.28 - 3.43	0.002
		**No **	374 (48.4)		399 (51.6)			
**PROM** **n** a **= 850 (271+579)**		**Yes **	180 (66.4)		91 (33.6)	2.70	1.99- 3.64	<0.001
		**No**	245 (42.3)		334 (57.7)			
**Aspect of amniotic fluid ** **n** a **= 850 (137+713)**		**Stained **	92 (67.2)		45 (32.8)	2.33	1.59 - 3.43	<0.001
		**Clear**	333 (46.7)		380 (53.3)			
**Place of delivery ** **n** a **= 846 (516+330)**	**Extra-hospital setting**			21 (67.7)	10 (32.3)	2.17	1.01 - 4.66	0.03
	**Hospital**			401 (49.2)	414 (50.8)			
**Mode of delivery ** **n** a **= 825 (260+ 790)**	**Caesarean section**			79 (30.4)	181 (69.6)	0.3078	0.22 - 0.42	<0.001
	**Vaginal**			346 (58.6)	244 (41.4)			

a number of informative files; PROM: Prolonged rupture of membranes; OR: Odds Ratio; CI: Confidence interval

**Table 5:** Distribution of factors associated with the neonate

**Parameter**		**Cases**	**Controls**	**OR**	**CI 95%**	***P. *** **value**
		**n (%)**	**n (%)**			
**Provenance ** **n** ^a^ **=846 (516+330)**	**YGOPH**	206 (39.9)	310 (60.1)	0.35	0.26 - 0.47	<0.001
	**Others**	216 (65.5)	114 (34.5)			
**Rank in the family ** **n** ^a^ **=804 (358+446)**	**1**	199 (55.6)	159 (44.4)	1.48	1.12 - 1.96	0.003
	**>1**	204 (45.7)	242 (54.3)			
**Type of pregnancy ** **n** ^a^ **=850 (66+784)**	**Multiple**	34 (51.5)	32 (8.5)	1.07	0.65 - 1.76	0.4
	**Single **	391 (49.9)	393 (50.1)			
**Sex ** **n** ^a^ **= 850 (454+396)**	**Male **	233 (51.3)	221 (48.7)	1.12	0.85 - 1.47	0.2
	**Female **	192 (48.5)	204 (51.5)			
**Gestational age (weeks) ** **n** ^a^ **=850 (398+452)**	**<37**	200 (50.3)	198 (49.7)	1.02	0.78 - 1.33	0.5
	**≥37**	225 (49.8)	227 (50.2)			
**Apgar score at 5** ^th^ ** minute ** **n** ^a^ **=850 (275+575)**	**< 7**	195 (70.9)	80 (29.1)	3.66	2.68 - 4.98	<0.001
	**≥ 7**	230 (40)	345 (60)			
**Birth weight (in grams) ** **n** ^a^ **=850 (404+446)**	**<2500**	221 (54.7)	183 (45.3)	1.43	1.09 - 1.88	0.005
	**≥2500**	204 (45.7)	242 (54.3)			

OR: Odds Ratio; CI: Confidence interval

It should be noted that this hospital is a mother and child referral hospital in Yaounde, the political capital of Cameroon. It receives patients from Yaounde and its environs as well as referrals from other parts of the country. 

 It is worth noting that the health care system in Cameroon has a pyramidal organization structure in three levels:

- Central level: with four 1^st^ category hospitals and three central hospitals,
- Intermediary level: constituted of 10 regional hospitals (3^rd^ category) in the 10 regions of Cameroon,
- Peripheral level: constituted of district hospitals (4^th^ category); subdivisional health centers (5^th^ category), integrated health centers (6^th^ category), and peripheral health posts (7^th^ category).

In this study the intra-hospital neonatal mortality was 9.83%. Higher rates were noted in Zimbabwe (19.3%)^[^^7^^]^, Kenya (31.5%)^[^^8^^]^, and Mali (38.8%)^[^^9^^]^. This is in contrast to the low rates observed in some developed countries: 0.38% and 0.35% in England and the United States respectively^[^^10^^,^^11^^]^. This could be due to poverty and ignorance prevailing in these countries which limit access to antenatal, intra-partum and postnatal care. The male predominance amongst the dead neonates has also been described by several authors^[^^12^^-^^14^^]^. Other studies noted a female predominance but still without any statistically significant difference^[^^8^^,^^15^^]^. Most deaths (93.4%) occurred in neonates admitted during the early neonatal period with 72.2% in the first 24 hours.

 Early neonatal deaths have also been noted to be higher than deaths in the late neonatal period in other studies^[^^8^^,^^9^^,^^12^^,^^13^^,^^16^^-^^21^^]^.

**Table 6:** Distribution of neonatal pathologies at admission

**Disorders**		**Cases**	**Control s**	**OR**	**CI 95%**	***P***
		**n (%)**	**n (%)**			
**Prematurity ** **n** ^a^ **=850 (364+486)**	**Yes **	181 (49.7)	183 (50.3)	0.98	0.75-1.29	0.5
	**No**	244 (50.2)	242 (49.8)			
**Neonatal sepsis ** **n** ^a^ **=850 (481+369)**	**Yes **	256 (53.2)	225 (46.8)	1.35	1.03 -1.77	0.02
	**No**	169 (45.8)	200 (54.2)			
**Neonatal asphyxia ** **n** ^a^ **=850 (225+625)**	**Yes**	159 (70.7)	66 (29.3)	3.25	2.34-4.51	<0.001
	**No**	266 (42.6)	359 (57.4)			
**Congenital defects ** **n** ^a^ **=850 (62+788)**	**Yes **	50 (80.6)	12 (19.4)	4.59	2.41-8.75	<0.001
	**No**	375 (47.6)	413 (52.4)			

OR: Odds Ratio; CI: Confidence interval

The early neonatal period is a delicate period because it is a period of transition between the intra-uterine and extra-uterine environment. At this age, the neonate is fragile with a weak immune status and body systems not quite adapted to the extra uterine environment, and therefore very susceptible to infections which can be fatal. 

 The average stay in hospital for the neonates who died was 5 days. Other authors noted 2, 4 and 8 days^[^^7^^,^^8^^,^^14^^]^.

*Maternal factors associated with labor and delivery:*

Prolonged rupture of membranes for more than 12 hours was strongly associated to the risk of death in our series. A similar finding has been noted by other authors^[^^22^^,^^23^^]^. Ruptured membranes for long periods expose the fetus to infections which can be a direct cause of death.

 Cesarean section was a protective factor against neonatal deaths. The babies born by C-section are systematically sent to neonatology for observation. Most of the babies may not have any pathology compared to those born per vaginally and admitted in the unit due to ill health. Another explanation may be the fact that YGOPH being a referral hospital receives cases requiring emergency cesarean section with indications being generally life-saving for the fetus, and also has C-section kits ready to manage emergency cases without delay. A similar finding was noted in other studies^[^^7^^,^^18^^]^. The higher mortality in neonates born vaginally may also be related to the fact that neonates born through cesarean sections were routinely admitted at the NNU for monitoring for at least two days, while those born vaginally were only admitted at the NNU when they presented signs and/or symptoms of disease. Similarly Ribeiro et al in Brazil noted a similar finding but with low birth weight neonates, and they postulated that this association (high neonatal mortality from vaginal deliveries) could be due to poor neonatal care following vaginal deliveries, and an inappropriate choice of the mode of delivery^[^^24^^]^.

 In this study, the number of prenatal visits did not statistically influence the risk of death, which goes to support the premise that it is the quality of these visits and not the number that influences pregnancy and eventually neonatal outcome. It is during these visits that malaria chemoprophylaxis, iron/folic acid supplements, tetanus vaccines are prescribed. Also routine investigations as u*rinalysis,* vaginal smears, HIV serology, syphilis testing, obstetrical ultrasounds and blood pressure monitoring are done. 

*Characteristics associated with neonates: *Most neonates who died came from other health facilities than those directly from the YGOPH maternity. Delivery in the YGOPH was a protective factor on the multivariate analysis with logistic regression. Other authors noted the same findings^[^^16^^]^. Transfer of neonates from one health facility or location to another in inappropriate conditions exposes the neonate to infections, hypothermia and delay in management, all factors which further reduce the chances of survival.

 Low birth weight was a risk factor. This observation has also been noted by several authors^[^^13^^,^^15^^,^^18^^,^^21^^]^. These neonates are at higher risk of asphyxia, sepsis, hypothermia and feeding problems. Besides, common diseases tend to be more severe and long-lasting in these neonates than in those with normal birth weights^[^^25^^]^.

 Other studies in Africa^[^^7^^,^^13^^,^^19^^,^^20^^,^^26^^,^^27^^]^, Iran^[^^15^^]^, Brazil^[^^18^^]^, and the Palestine^[^^28^^]^, also noted that low birth weight was a risk factor for neonatal mortality.

 Prematurity is one of the main causes of low birth weight, but in this study it was not found to be a risk factor for neonatal deaths. A likely explanation for this could be due to the fact that the cases and controls were paired for gestational age plus or minus two weeks.

 A low Apgar score <7 at the 5th minute was strongly associated with neonatal mortality. A low Apgar score has been shown to be correlated with neonatal mortality^[^^29^^]^. However the interpretation of the Apgar score has limitations especially in premature neonates, in whom the tone, color and reflexes partially depend on the physiological maturity of the newborn^[^^29^^,^^30^^]^. Our finding is similar to those of Eloundou^[^^17^^]^, and Chiabi et al^[^^31^^]^, whereas others noted that a low Apgar score at both the 1^st^ and 5^th^ minute was a risk factor for neonatal mortality^[^^14^^]^.

*Neonatal pathologies on admission: *Neonatal sepsis, birth asphyxia and congenital malformations were statistically associated with neonatal mortality. However the main causes of deaths in descending order were: neonatal sepsis, complications from prematurity, birth asphyxia and congenital malformations. The same causes were noted by Lawn et al^[^^32^^]^.

 The high mortality from congenital malformations despite the presence of a pediatric surgery unit at the YGOPH, could be explained by the frequent lack of antenatal diagnosis, lack of a well-equipped ambulance transport system during referral, the delay in surgical procedures, poverty, and an inadequate surgical, anesthetic and neonatal resuscitation platform^[^^33^^]^. Other authors also observed a significant association between congenital malformations and neonatal mortality^[^^7^^,^^14^^,^^18^^,^^21^^,^^34^^]^.

## Conclusion

This study noted that neonatal mortality was influenced by maternal factors (prolonged rupture of membranes for more than 12 hours, cesarean section) and neonatal factors (low birth weight, Apgar score <7 at the 5th minute, congenital malformations and place of delivery).

 Sensitization of pregnant women, through information-education-communication, on the importance of antenatal care for early detection and appropriate management of high-risk pregnancies could help reduce neonatal mortality. The technical platform for proper management of congenital malformations in this setting should be improved, and as well as building capacity of health personnel to adequately manage high risk neonates with infections, prematurity, asphyxia and low birth weight.
